# Experimental identification of wheel-surface model parameters: various terrain conditions

**DOI:** 10.1038/s41598-022-19829-7

**Published:** 2022-09-26

**Authors:** Tomasz Czapla, Marcin Fice, Roman Niestrój

**Affiliations:** 1 Department of Applied Mechanics, Silesian University of Technology, Faculty of Mechanical Engineering, Akademicka 2A, 44-100 Gliwice, Poland; 2 Department of Electrical Engineering and Computer Science, Silesian University of Technology, Faculty of Electrical Engineering, Akademicka 2A, 44-100 Gliwice, Poland

**Keywords:** Engineering, Electrical and electronic engineering, Mechanical engineering

## Abstract

Since the wheel interaction with a certain terrain cases (asphalt, concrete) are known and well described in case of straightforward motion and non-slip and slip cornering conditions, the skid-steered wheeled vehicles case needs to be analyzed. Side-slip for various attack angle has to be investigated. The main area of interest of research that is shown in the project is energy demand calculation of skid-steered wheeled vehicles in various terrain conditions. Certain cases of all-electric vehicles with individual electric motors per wheel demand a precise assessment of longitudinal and lateral forces in order to perform the fully controlled turn. Experimental stand designed and developed by authors allows to test the wheel-surface interaction for various terrain conditions and different driving directions. Test data were acquired for dry and wet sand and granite pavement. Traction and side forces were acquired and used to identify the wheel-soil interaction model parameters for unpropelled wheel. Results in a form of time series including longitudinal and lateral forces show the relation between attack angle, load and surface conditions in terms of stick and slip phenomenon that is essential for skid-steering dynamics calculations. Measurement results are then used for calculation of longitudinal and lateral forces coefficients as a function of attack angle and vertical load. Test were performed in natural environment, thus they are affected by changeable conditions. Multiple runs are used for elimination of that influence. Described experiments are a part of the project that includes results generalization using test validated FEM model. Described work is not intended to develop new ground-tire interaction models, it is focused on numerically efficient traction effort calculation method for various conditions including passive mode—unpropelled wheel.

## Introduction

All-terrain vehicles, especially unmanned and autonomous machines are optimized in order to minimize dimensions and weight. As a consequence, less complex transmission and steering systems are used in design of small, medium and heavy unmanned vehicles. The most common solution are elastic suspension and skid steering using electric or hydraulic traction motors. In case of off-road vehicle with electric propulsion system vital parameter is energy reservoir volume. For electric propulsion system with high overload abilities it is vital to assess the medium continuous torque and power and also the maximum performance parameters that will allow to tune the power station, energy storage system and propulsion motors correctly. Proper analysis of power consumption in various terrain and exact mission definition will allow to optimize the battery system what will allow to use modular design batteries configured according to mission demands. In order to assess the energy demand it is essential to create the universal and quick numerical method for energy usage prediction.

The work described in this paper is a part of the project focused on development an universal methodology for design, optimization and analysis of modern propulsion system for various types of vehicles and terrain conditions. Since the on-road vehicle behavior and performance is well-described, there is a lack of accessible knowledge concerning off-road performance of various types of vehicles. Methods of assessing traction effort are based on complex and often inadequate theoretical models on one hand and experimental testing of certain type of vehicles on the other. The methodology proposed in this paper is a combination of experimental, theoretical and numerical methods that will allow to perform fast traction effort calculation with acceptable accuracy. The most important aspect of the research was prediction of lateral and longitudinal forces for unpropelled wheel. As it was observed in previous research performed for tracked vehicles—there is possibility to recuperate energy from internal track and—as it will be investigated in further research—for wheeled vehicles. The simplest and the most demanding in terms of torque generated by electric motors will be zero-turn. In case of various and fully controlled turns there is vital to investigate the resistant forces for unpropelled wheel for different attack angles and calculate possible energy recuperation level.

## State-of-art analysis

Dynamics of wheeled vehicles in road conditions has been widely described and various model are proposed to describe the wheel-road behavior. The most important phenomenon is stick–slip mechanism that was considered with using various friction models^1^. Models also are used to assess tire deformation in various cases: vertical load in a steady-state condition, longitudinal and lateral (transverse) force. Analytical and numerical modelling methods in most cases neglect wheel deformation under the side-load despite its contribution in rolling resistance increase^2^. Empirical models, FEM methods and spring-damper wheel models can be used to achieve more accurate results^3^. As the more complex approach tire-ground interaction were based mainly on experimental soil parameters testing. Cone testing was used for soil shear, tension and extension strength measurement^4,5^. Ground testing technology was first introduced by Bekker^6^. Bekker method was based on two tests: plate sinkage test and shear test. Plates with normalized dimensions were used to measure the sinkage and shear rings or plates to measure the shear. As the result, relationship between pressure and ground deformation is calculated as it is indicated by sinkage

1 $$\sigma =\left(\frac{{k}_{c}}{B}+{k}_{\varphi }\right) \cdot {z}^{n},$$

2 $$\tau =\left({k}_{cohesion}+\upsigma \cdot \text{sin}\varphi \right) \cdot \left(1-{e}^{j/k}\right),$$

where, $\sigma$ is the normal pressure, *k*_*c*_*, k*_*ϕ*_*, n* are soil properties parameters, *z* is the soil sinkage, *B* is the tire width, *ϕ* is the free angle of shearing soil resistance, *τ* is the shear stress, *k*_*cohesion*_ is the soil cohesion, *j* is the shear displacement.

Bekker^7^ model based was the first complex method of stress calculation in the tire—soil contact area and beneath. Bekker equation allows to calculate the normal pressure as a function of the sinkage (Eq. 1), modified Coulomb equation (Eq. 2) is used to calculate the shear stress with taking into account parameters as shear displacement, cohesion and soil shear deformation^8^.

The Bekker model and other early approaches neglect the tire deformation influence on wheel—surface dynamics due to wheel rigidity assumption. Lately developed models allow to take into account the tire deformation (Schmid^9^). Modern research is focused on Finite Element Analysis.

Hydraulic and electric propulsion systems allow to drive each wheel individually, so that precise torque distribution is available. There are several approaches for wheel-ground tests with using one wheel testbed^10,11^. Analyzed researches include propelled wheel testbeds^12^ with attack angle control in laboratory conditions. There are also locked and unpropelled wheels cases described and tested^12,13^. The aim of the research is to find the most efficient method for electric all-terrain locomotion that could be implemented in vehicle control unit. As it was described by Flippo and Miller^14^ there is a need of single-wheel stands testing—especially in case of research described in this paper based on a full-size wheel. Improvement of resistance loads calculation methods is essential for UGV design in order to optimize control systems and accuracy taking into account minimalization of energy demands for propelling vehicles^15^.

## Methodology description

**Figure 1:**
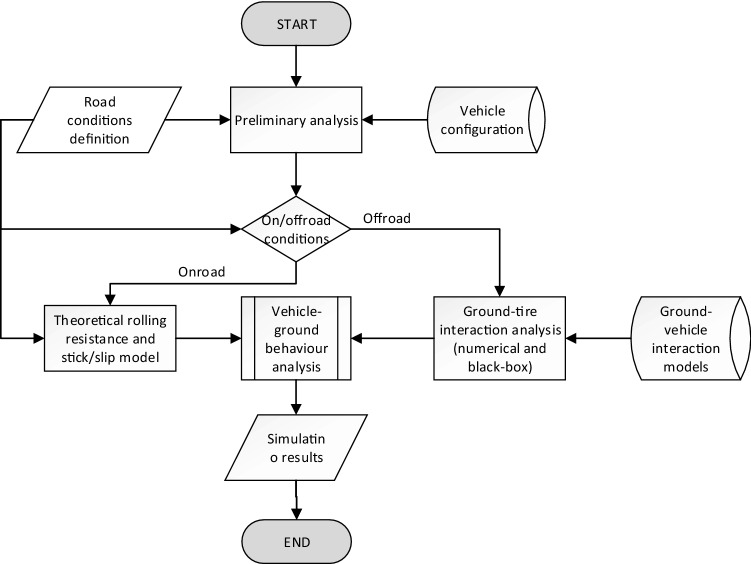
Ground tire interaction model.

The method, described in the paper is a part of the project focused on providing an accurate model of traction effort for various vehicle configurations. There are two databases containing road conditions and vehicle configuration. Road conditions include the terrain type, demanded path, speed and performance of the vehicle. Vehicle configuration includes mass, steering mechanism, number of wheels and its design. Based on preliminary analysis, the model would be selected. For simple cases of on-road performance, theoretical model will be selected. For ground off-road conditions, model based on experimental testing and FEM analysis will be applied. For various conditions and different wheel configuration traction forces will be calculated. Model data are validated with the field testing results (Fig. 1).

Based on field test and numerical analysis results, vehicle rolling resistance and, especially for skid-steered vehicle turning moment will calculated. Since the authors are focused on simplified design of unmanned vehicles, skid-steering is taken into account. That allows to take benefits from abilities of electric propulsion system: individually propelled wheels, zero-turn ability, high overload ratio.

**Figure 2:**
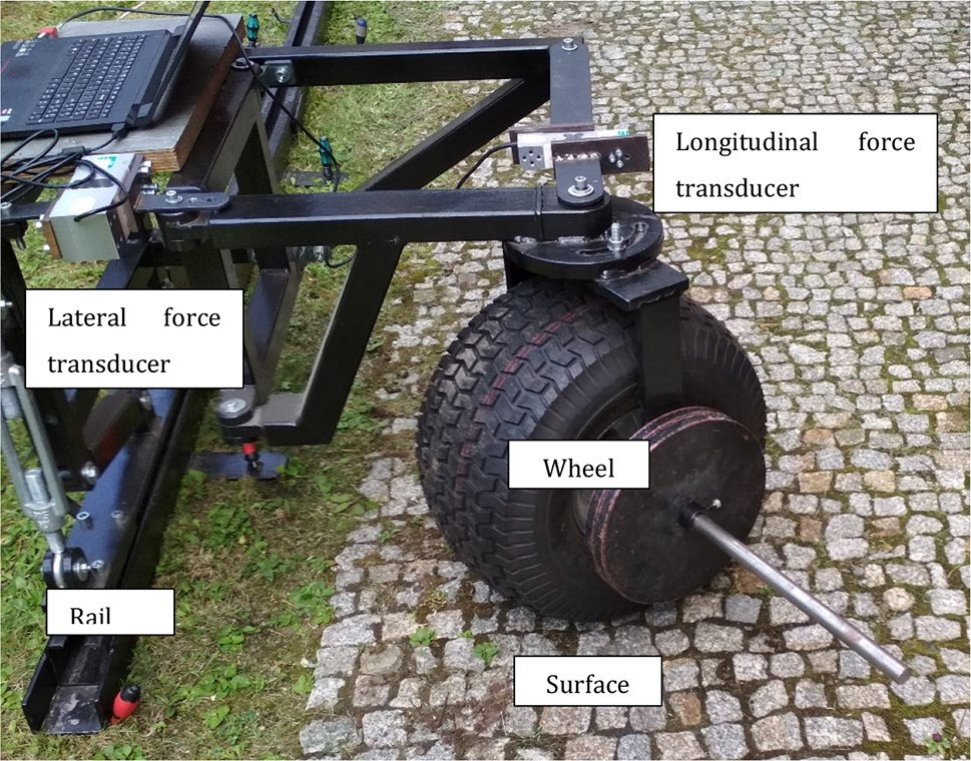
Experimental stand.

As a representation of each wheel during the tests the laboratory stand was designed and used for simulation of different geometrical and road conditions. The wheel movement was performed by external propulsion system. In case of presented tests there was no electric motor propelling the wheel. The construction contains two basic elements: rail and frame. Rail is responsible for keeping the proper direction of wheel motivation and frame transfers the load from the wheel to the ground via force transducers. The transducers are connected to the rods what allows to isolate two load measuring directions: longitudinal and lateral defined in frame coordination system. It is possible to turn the wheel in vertical axis to achieve different propulsion directions (Fig. 2).

Experimental stand allows to achieve various wheel performance parameters configuration for different and specific ground conditions. Angle of attack (angle between wheel vertical symmetry plane and motion direction) could be changed from 0° to 90°. Other parameters that could be changed are: wheel load, tire pressure. The stand is a mobile construction and it can be used in the field to measure the forces in natural conditions. Unique design of the stand allows to measure longitudinal and lateral force in the moving cart coordinate system. For testing the off-road threaded wheel size 20 × 10.00–8″ was used. On the Fig. 4 wheel geometry during the skid-steering maneuver and measured forces are shown.

Based on direct measurement it is possible to calculate forces related to the geometry of the wheel. F_long_ and F_lat_ forces are the projection of F force resultant of F_long_ and F_lat_ on the wheel coordinate system. F_long wheel_ and F_lat wheel_ forces can be derived with using Park transform assuming that x-axis defines lateral forces in coordinate system attached to the frame.

**Figure 3:**
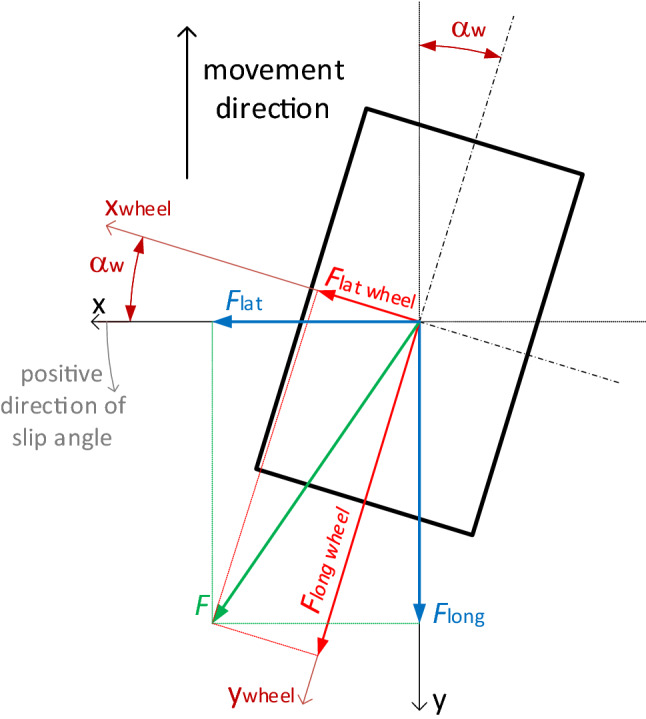
Geometrical model of the wheel, top view. x–y—coordinate axes relate to the movement direction, x_wheel_–y_wheel_—coordinate axes related to the wheel geometry, F_long_—measured longitudinal force related to the movement direction, F_lat_—measured lateral force related to the movement direction, F—the resultant force related to coordinate axes of movement direction, F_long wheel_—longitudinal component of the force related to geometrical wheel axes, Flat wheel—lateral component of the force related to geometrical wheel axes, α_w_—slip angle (between movement direction and wheel coordinate system)^16^.

Related to the Fig. 3 total longitudinal and transverse forces related to geometrical axes of the wheel can be expressed as below:

3 $${F}_{\text{long wheel}}={F}_{\text{long}} \cdot \text{cos}\left(-{\alpha }_{\text{w}}\right)-{F}_{\text{lat}} \cdot \text{sin}\left(-{\alpha }_{\text{w}}\right),$$

4 $${F}_{\text{lat wheel}}={F}_{\text{long}} \cdot \text{sin}\left(-{\alpha }_{\text{w}}\right)+{F}_{\text{lat}} \cdot \text{cos}\left({-\alpha }_{\text{w}}\right).$$

**Figure 4:**
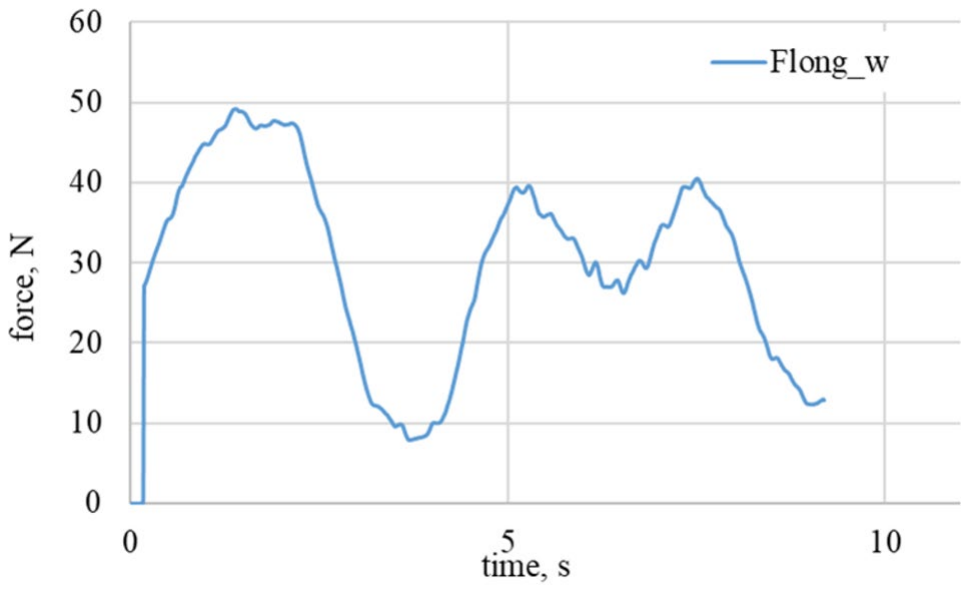
Longitudinal and lateral forces related to the wheel geometry for α_w_ = 0° for granite surface.

For the case shown in the Fig. 4 for chosen coordinate system force F_lat wheel_ has negative value. In case of maximum wheel turn angle, α_w_ = 90°, xwheel axis is parallel to y axis and ywheel axis will be consequently parallel to x-axis, so:

5 $${F}_{\text{long wheel}}=-{F}_{\text{long}},$$

6 $${F}_{\text{latwheel}}={F}_{\text{lat}}.$$

Resultant longitudinal force F_long wheel_, is responsible for generation of traction torque. It can be assumed that F_long wheel_ is a traction effort force. Each wheel generates the resistant force caused by slip caused by turning moment allowing to perform the turn in case of skid-steered vehicle.

## Results analysis

Tests were carried out for 4 attack angles α_w_ = 0°, 30°, 60° and 90°. The pressure in the tire was 0.15 MPa, and wheel vertical load was 80 kg. The wheel was propelled on the snow and granite surface with the speed of 0.5 m/s. Results for steady-state conditions are shown in graphs below.

**Figure 5:**
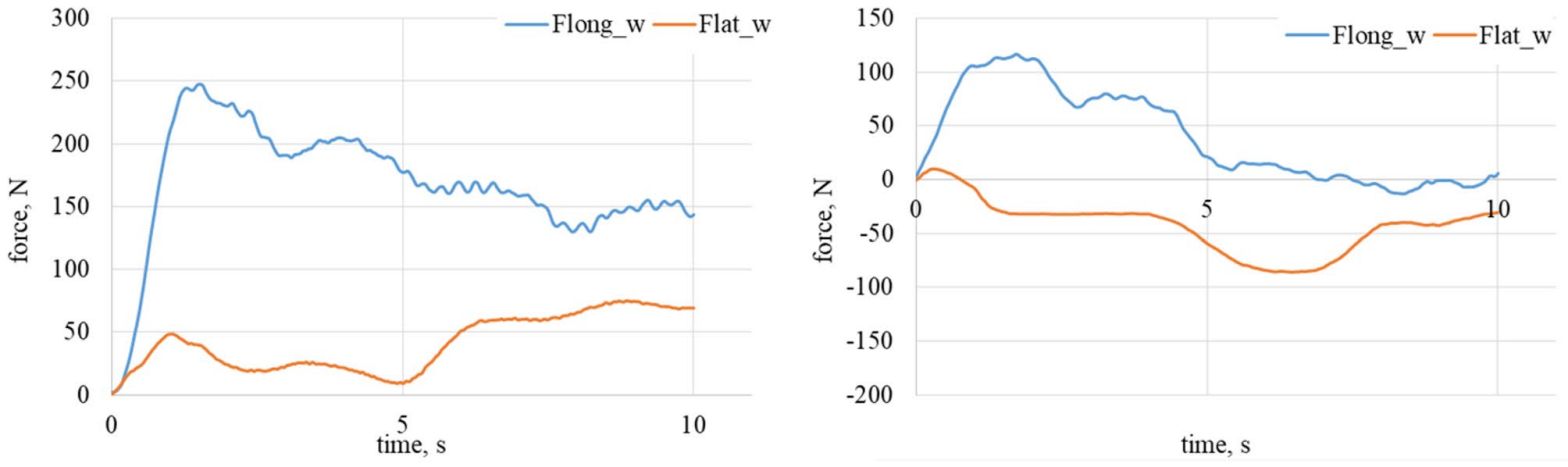
Longitudinal and lateral forces related to the wheel geometry for α_w_ = 0°. On the left waveform: loose snow; on the right waveform: compacted snow.

**Figure 6:**
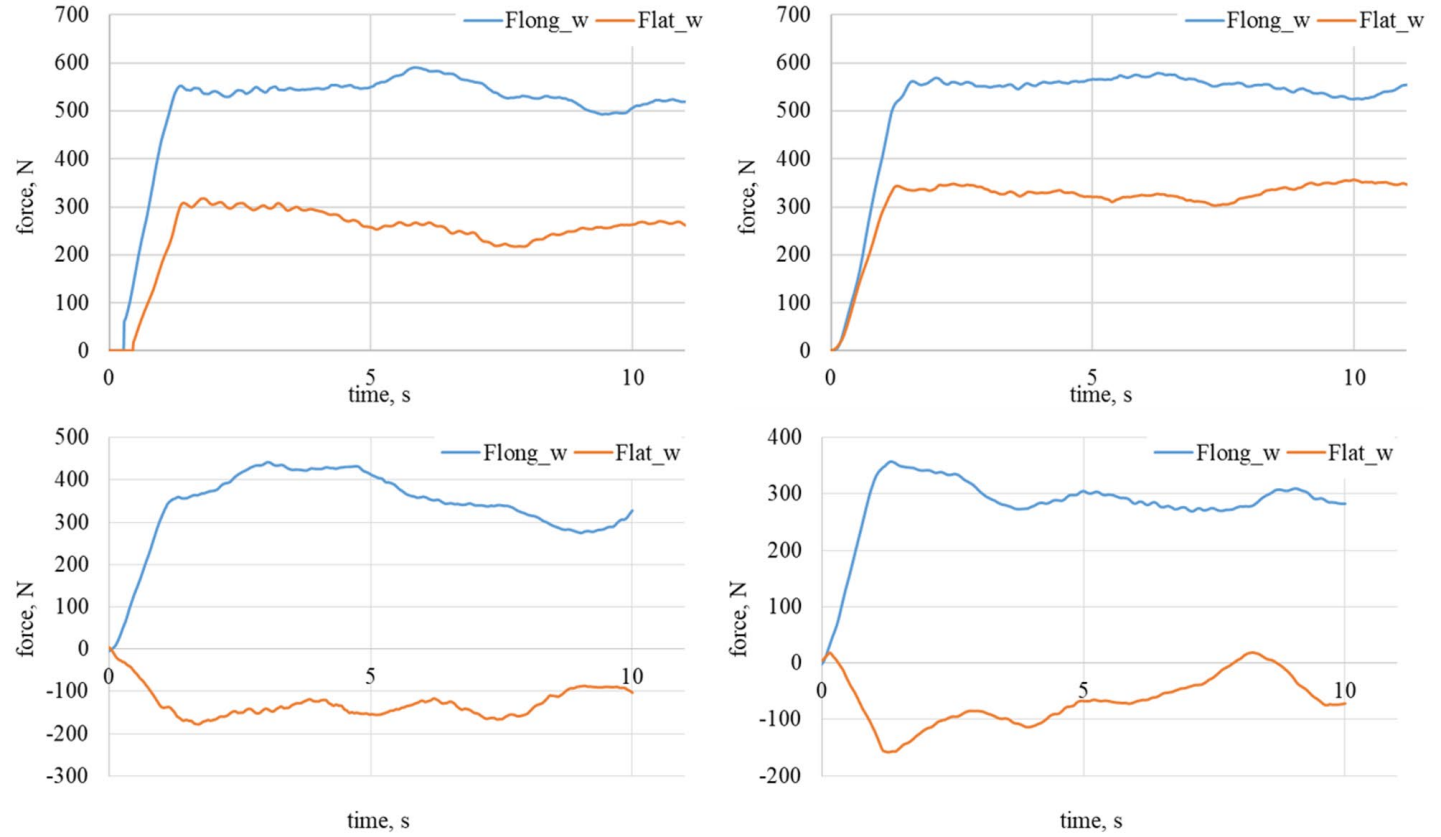
Longitudinal and lateral forces related to the wheel geometry α_w_ = 30°. On the top left waveform: dry granite; on the top right waveform: wet granite. On the bottom left waveform: loose snow; on the bottom right waveform: compacted snow.

**Figure 7:**
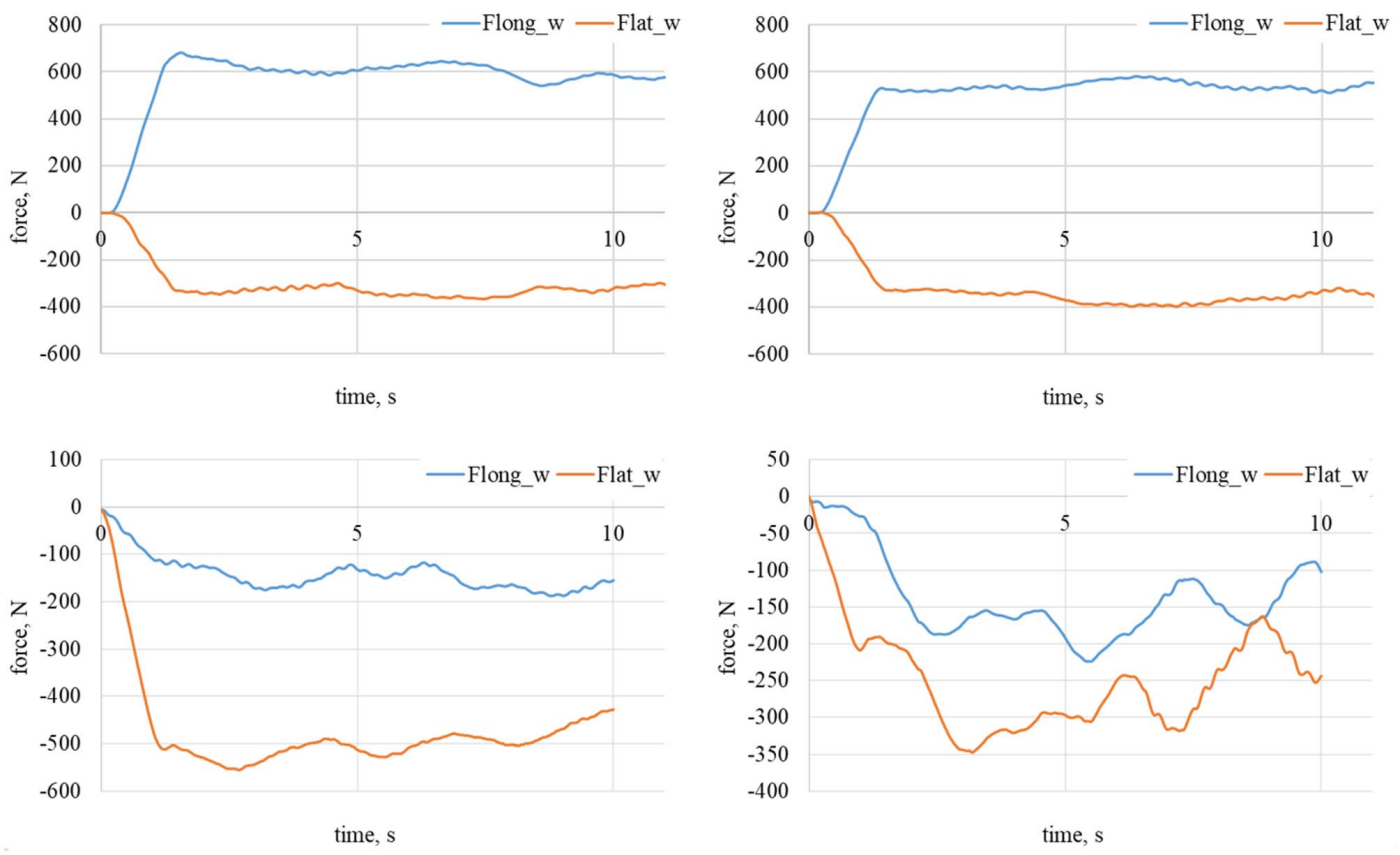
Longitudinal and lateral forces related to the wheel geometry for α_w_ = 60°. On the top left waveform: dry granite; on the top right waveform: wet granite. On the bottom left waveform: loose snow; on the bottom right waveform: compacted snow.

**Figure 8:**
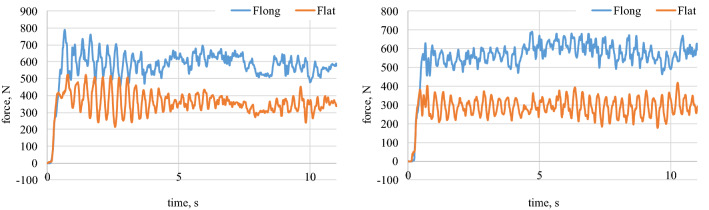
Longitudinal and lateral forces related to the wheel geometry for α_w_ = 60°. On the left waveform raw data for dry granite; on the right waveform raw data for the wet granite.

**Figure 9:**
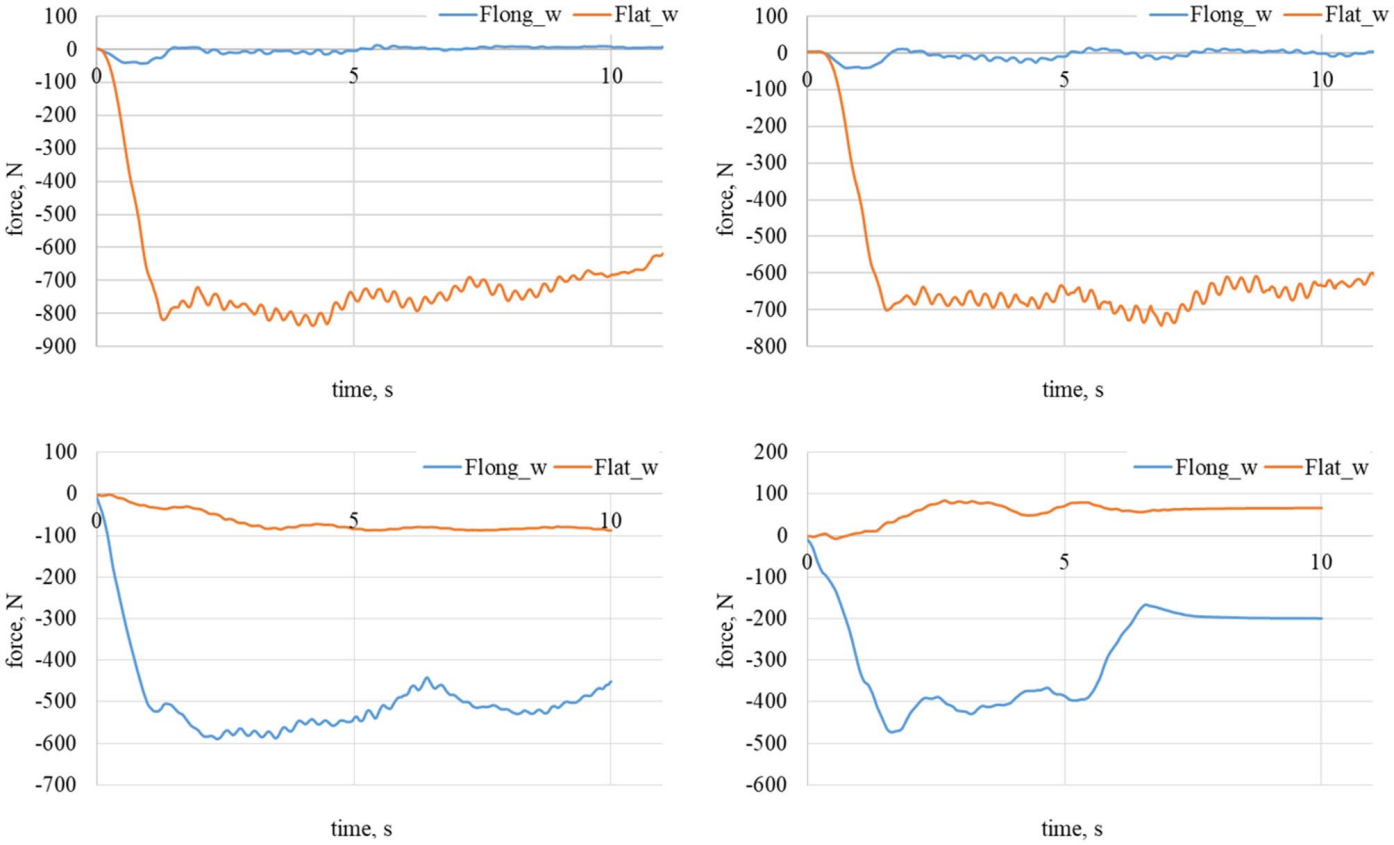
Longitudinal and lateral forces related to the wheel geometry for α_w_ = 90°. On the top left waveform: dry granite; on the top right waveform: wet granite. On the bottom left waveform: loose snow; on the bottom right waveform: compacted snow.

**Figure 10:**
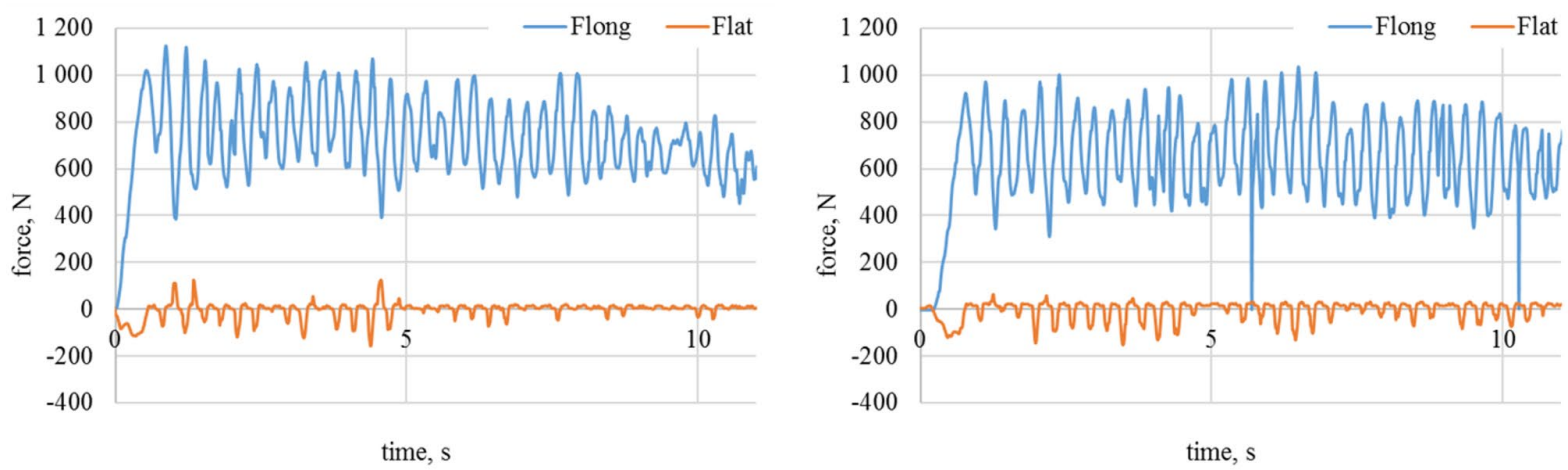
Longitudinal and lateral forces related to the wheel geometry for α_w_ = 90. On the left waveform raw data for the loose snow; on the right waveform raw data for compacted snow.

On the Figs. 4 and 5 the least demanding cases are shown. Attack angle is equal to 0, so that lateral forces should achieve the minimum. There could be observed variation of longitudinal forces caused by the surface imperfections. Further experiments shown in Figs. 6, 7, 8, 9 and 10 show the lateral force increase due to angle of attack rising. In case of 90° there is a major contribution of longitudinal force and lateral force is equal to 0. Figures 8 and 10 show the raw data from the experiments. As it could be observed there are periodical stick–slip phenomena that have to be further investigated.

## Wheel-surface model formulation

The approach proposed in previous works and this article leads to empirical traction effort calculation model for skid-steered vehicle. The model will allow to calculate of yaw moment for skid-steered wheeled vehicle on various surfaces and in various terrain conditions. The first step was calculation of resistance forces that will allow to calculate the traction force for each wheel.

Input parameters for the model are^16^.

- Slip angle,
- Normal force (wheel vertical load),
- Wheel pressure,
- Ground pattern.

Output parameters of the model.

- Longitudinal force—contribution to the traction resistance of the vehicle,
- Lateral force—contribution to the traction resistance of the vehicle,
- Longitudinal wheel force—generates traction resistance for each wheel,
- Lateral wheel force—generates bending moment for the wheel and loads for the suspension system.

**Figure 11:**
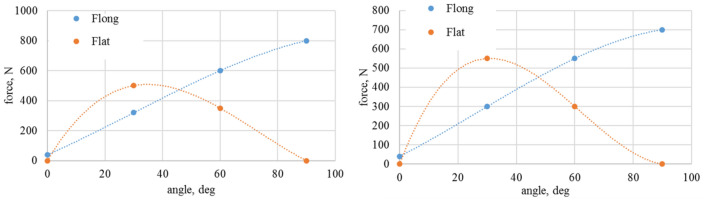
Values of longitudinal and lateral forces depending of the slip (attack) angle—dry granite.

**Figure 12:**
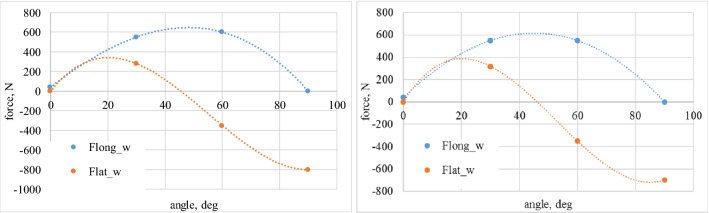
Values of calculated longitudinal and lateral forces depending of the slip (attack) angle—wet granite.

**Figure 13:**
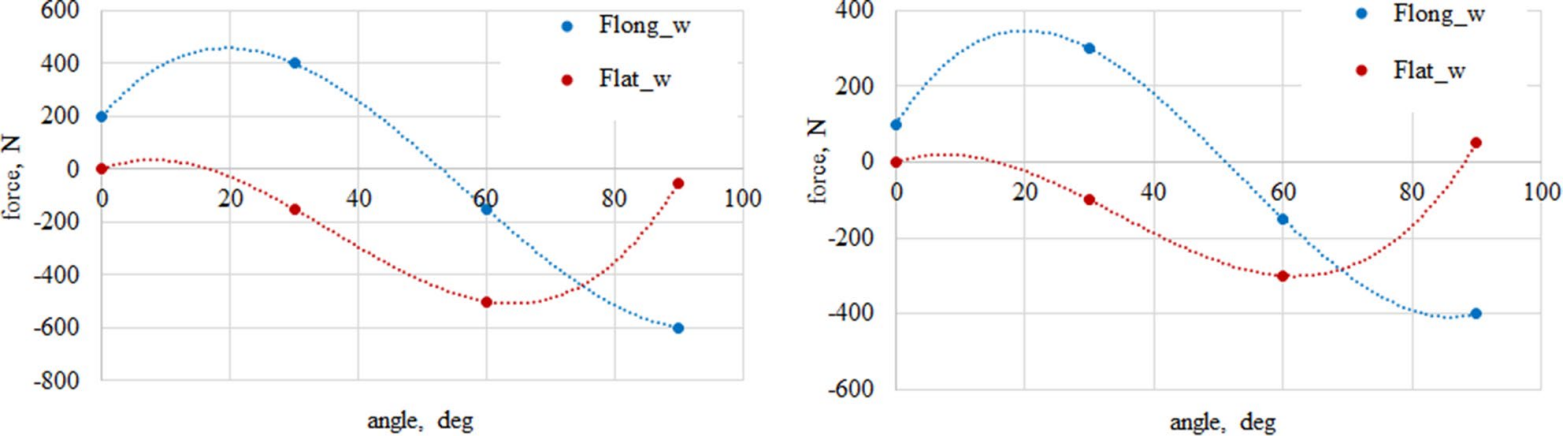
Values of longitudinal and lateral forces depending of the slip (attack) angle—loose snow.

**Figure 14:**
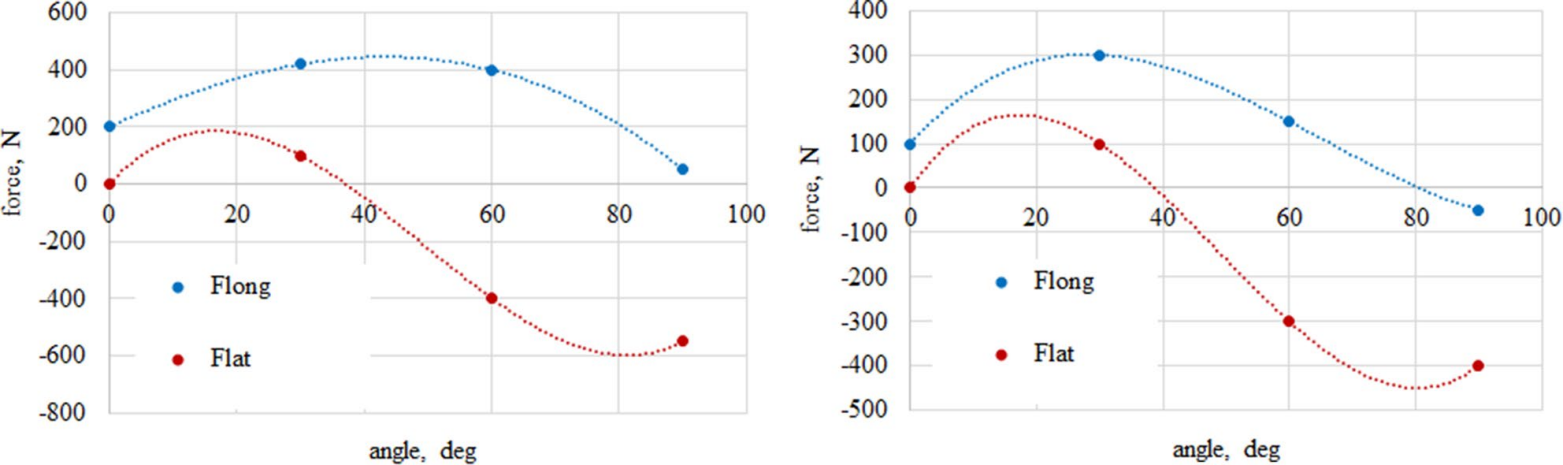
Values of calculated longitudinal and lateral forces depending of the slip (attack) angle—compacted snow.

Model is based on experimentally derived load patterns for loads acting on each vehicle wheel. Figures 11, 12, 13 and 14 show an averaged test results in the function of slip angle for performed test conditions.

Resultant traction resistance could be derived as it is shown in Eq. (7)

7 $${F}_{t}={g}_{m} \cdot {p}_{t} \cdot {f}_{t}\left({\alpha }_{w}\right),$$

where, *f*_t_ is the traction resistance coefficient, *p*_t_ is the tire pressure coefficient, *g*_m_ is the wheel load.

In order to generalize results, two coefficients are introduced: longitudinal and lateral resistance coefficient (Eqs. 8, 9).

8 $${f}_{long}=\frac{{F}_{long}}{G}-longitudinal\;wheel\;resistance\;coefficient,$$

9 $${f}_{lat}=\frac{{F}_{lat}}{G}-lateral\;wheel\;resistance\;coefficient,$$

where, *G* is the vehicle weight (mg).

## Conclusions

The method presented in the article is a part of the project focused on approximate calculation of traction forces for unmanned vehicles in case of unpropelled wheels mode.

Test were performed in natural conditions and were affected by the snow discontinuity, pavement geometrical imperfections and weather conditions. It is recommended that test should be done in two separated ways: in laboratory fully controlled environment and in field with using the 4–6 wheeled vehicle. Future work will be focused on the design of laboratory facility and test vehicle.

FEM model validated with experiment results will be used to generalize the model. Presented results are contribution to general wheel-ground interaction model that is developed by authors.

The methodology is not intended to develop new ground-tire interaction models, it is focused on numerically efficient traction effort calculation method.
